# Selenium and its nanoparticles modulate the metabolism of reactive oxygen species and morpho-physiology of wheat (*Triticum aestivum* L.) to combat oxidative stress under water deficit conditions

**DOI:** 10.1186/s12870-024-05282-3

**Published:** 2024-06-19

**Authors:** Mirza Hasanuzzaman, Md. Rakib Hossain Raihan, Ayesha Siddika, Kirti Bardhan, Md. Sarwar Hosen, P. V. Vara Prasad

**Affiliations:** 1 Department of Agronomy, Faculty of Agriculture, Sher-e-Bangla Agricultural University, Dhaka, 1207 Bangladesh; 2 Department of Basic Sciences and Humanities, Navsari Agricultural University, Gujarat, India; 3 Institute of Seed Technology, Faculty of Agriculture, Sher-e-Bangla Agricultural University, Dhaka, 1207 Bangladesh; 4 Department of Agronomy, Kansas State University, Manhattan, KS USA

**Keywords:** Abiotic stress, Climate changes, Plant water relations, Oxidative stress, Nanoparticles, Trace elements

## Abstract

**Background:**

Wheat (*Triticum aestivum* L.) is one of the most important cereal crop species worldwide, but its growth and development are adversely influenced by drought stress. However, the application of trace elements is known to improve plant physiology under water-limited conditions. In this study, the effects of drought stress on wheat plants were investigated, with a focus on potential mitigation by foliar application of selenium nanoparticles (Se(np)) and sodium selenate (Na_2_SeO_4_). The experiment was conducted in a net house using a completely randomized design with four replications. The treatments involved three levels of drought stress (mild, moderate, and severe) started at 30 days after sowing (DAS), with foliar sprays of Se(np) and Se (both 25 µM) initiated at 27 DAS and repeated 4 times at 7-day intervals until 55 DAS.

**Results:**

Drought stress significantly reduced plant growth, whereas Se(np) and Se sprays enhanced it. Drought stress induced chlorophyll degradation, increased malondialdehyde and hydrogen peroxide levels, impaired membrane stability, and caused electrolyte leakage. Severe drought stress reduced the levels of antioxidants (e.g., proline, ascorbate, and glutathione by 4.18-fold, 80%, and 45%) and the activities of antioxidant enzymes (ascorbate peroxidase, dehydroascorbate reductase, and others). Conversely, treatment with Se(np) and Se restored these parameters, for example, 1.23-fold higher total chlorophyll content with Se(np) treatment, 26% higher APX activity with Se treatment, 15% lower electrolyte leakage with Se treatment in wheat plants under severe drought stress. This Se-associated enhancement facilitated rapid scavenging of reactive oxygen species and reduced methylglyoxal toxicity, thereby diminishing oxidative stress and positively affecting the morphophysiological and biochemical responses of the plants under drought.

**Conclusions:**

Drought-stressed wheat plants exhibited reductions in physiological processes, including water uptake and photosynthetic activity. However, Se(np) and Se applied at 25 µM mitigated the detrimental effects of drought. The application of Se(np) was notably more effective than the application of Se in mitigating drought stress, indicating the potential of the application of Se(np) as a sustainable agricultural practice under water-limited conditions.

**Supplementary Information:**

The online version contains supplementary material available at 10.1186/s12870-024-05282-3.

## Background

Global crop production is mostly threatened by water scarcity, and climactic variabilities signal that seasonal drought in crop fields will continue to be a major constraint limiting the future food and nutritional demands of the growing world population [1, 2]. Wheat (*Triticum aestivum* L.), which is grown globally on 220.76 million ha [3], is considered a staple crop worldwide, but wheat yields are severely limited by abiotic stresses, such as seasonal drought [4]. The Intergovernmental Panel on Climate Change (IPCC) emphasizes that more frequent and severe drought occurrences are anticipated as a result of climate change. This raises the threat to wheat production worldwide and more specifically in drought-prone areas. Wheat yield may be severely reduced by drought stress, particularly during critical growth stages for example flowering and grain filling stage. Yield losses of up to 50% or more are possible in cases of severe drought. The IPCC [5] projects that wheat yields would further fall due to the predicted 1–4 °C rise in global temperature by 2100. The limitations imposed by drought arise from biochemical changes that disrupt cellular homeostasis and trigger the overproduction of reactive oxygen species (ROS) in chloroplasts in response to stomatal closure and imbalances in photochemistry. ROS accumulation in plant cells causes lipid peroxidation and nucleic acid damage, inhibits enzyme activities, promotes electrolyte leakage, and dysregulates carbon metabolism [6].

Under nonstress conditions, ROS are effectively managed by intrinsic antioxidant enzymes, such as superoxide dismutase (SOD), catalase (CAT), and peroxidase (POD); consequently, improvements in the activities of these enzymes during drought improve crop drought tolerance. Glyoxalase enzymes work alongside to negate the negative effects of overproduced methylglyoxal (MG) under drought stress. Studies have suggested that genotypes that show early and high activities of antioxidant defense compounds also show less hydrogen peroxide (H_2_O_2_) accumulation and lipid peroxidation, along with greater drought tolerance [7]. These findings suggest that the selection of plants with these traits would enable the breeding of drought-tolerant varieties; however, selective breeding of drought-tolerant wheat varieties is limited by the multifactorial nature of drought and variations in the intensity and duration of drought in the field, as well as the narrow genetic base and genome size of wheat [8]. For these reasons, crop management approaches, such as the foliar application of selenium (Se), are gaining importance as on-time protocols for the management of drought and other abiotic stresses in different crops [9, 10]. In addition to providing timely drought management, these approaches also overcome the limitations of attempting to breed genotypes tailored to specific soil conditions.

The application of Se at low concentrations improves ROS scavenging in plant cells by enhancing intrinsic enzymatic and nonenzymatic antioxidant systems, thereby improving crop growth characteristics [10, 11]. However, recent studies have emphasized the benefits of using nanoparticles (NPs) as carriers of important metals, as NPs are readily taken up by plants and are able to cross cellular membranes. Foliar applications of different metal NPs have been shown to improve chlorophyll (Chl) contents, osmolyte levels, and antioxidant activities in different crops [10, 12]. Moreover, in addition to protecting against accumulated ROS, NP treatments also activate plant antioxidant defenses and induce the synthesis of protective secondary metabolites [13]. Notably, nanoparticles containing selenium (Se(np)) have been demonstrated to increase antioxidant levels and alter ROS signaling in plants [10, 11]. However, information is scarce regarding the potential benefits of Se and Se(np) application on the amelioration of drought effects or their influence on various physiological and biochemical parameters. Therefore, the aim of the current study was to assess the effects of foliar applications of Se and Se(np) on growth and antioxidant levels (both enzymatic and nonenzymatic) in wheat plants under drought conditions.

## Materials and methods

### Experimental setup, treatment, and design

Seeds of wheat (*Triticum aestivum* L.) cv. BARI Gom 30 were obtained from the Bangladesh Agricultural Research Institute (BARI) and healthy and uniformly sized seeds were selected for sterilization before sowing in plastic pots (14 L) filling up with sandy loam soils free of stubbles. Soil was air dried before preparation to kill weeds and insects. The experiment was carried out in a greenhouse with average day and night temperatures of 24.5 °C and 15.4 °C, respectively, and the relative humidity was approximately 52.5%. Following the BARI recommendations [14], fertilizer was added during soil preparation, and 13.5 kg of soil was used to fill each pot.

Selenium nanoparticles were prepared following the method of El Lateef Gharib et al. [15], who used ascorbic acid as a reducing and stabilizing agent to reduce sodium selenate (Na_2_SeO_4_). The reaction was initiated by slowly adding ascorbic acid powder (1.5% w/v) to a 10 mM aqueous Na_2_SeO_4_ solution [16] and stirring at room temperature for 15 min. The reaction mixture was then left to stand until the solution turned light orange. The absorbance was measured using a spectrophotometer at 300 nm at 1 h and again every 15 min to confirm that no further changes occurred in the absorbance, indicating that the reaction was complete. When the color change was completed, the residue dried in a hot air oven at 200 ° C for 72 h and then calcined at 450° C in a Muffle furnace and preserved at 45 °C. The particles were characterized using scanning electron microscopy (SEM) and transmission electron microscopy (TEM) which is presented in the Supplementary file (Fig. S1). The solution was diluted to 25 µM with distilled water and used as the foliar Se(np) spray (pH 3.4).

Three levels of drought (50%, 25%, and 12.5% field capacity, denoted as mild [D_1_], moderate [D_2_], and severe [D_3_] drought, respectively) were maintained from 30 days after sowing (DAS) until physiological maturity. Field capacity was maintained by controlling the soil moisture based on the preliminary experiment and a soil moisture meter (Model no. WH0291) was used to measure the soil moisture level. The control plants were irrigated as needed. Spraying of sodium selenate (Na_2_SeO_4_; FujiFilm Wako Pure Chemical Corporation, Osaka, Japan) and Se(np) (both 25 µM) was initiated at 27 DAS, and repeated 4 times until 55 DAS at 7-day intervals. The study was conducted as a completely randomized design with four replications. Upon completion of the treatment, different morphological, physiological, and biochemical data were collected at 60 DAS.

### Determination of plant height and biomass

Five plants were randomly selected from each treatment, and plant height was measured using a scale from the bottom of the plant to the tip of the longest leaf. The findings were averaged and are presented in centimeters (cm). The fresh weight (FW) of randomly selected plants from each set of treatments was determined. The entire plant was harvested, dust particles were removed, and the plant was carefully weighed using a digital balance. Each plant was air-dried and then transferred to an 80 °C oven to dry for 72 h, followed by dry weight (DW) measurements. The average FW and DW values are presented as g plant^− 1^.

### Determination of chlorophyll levels

Chlorophyll content was estimated as per the method described by Arnon [17] by placing 0.25 g of chopped leaf tissue into 10 mL of 100% ethanol and boiling it in a water bath until the tissues turned white. Then spectrophotometric measurements were taken at 663, 645, and 470 nm and Chl *a*, Chl *b*, and Chl (*a* + *b*) pigments contents were calculated.

### Determination of lipid peroxidation rates

The leaf malondialdehyde (MDA) concentrations were quantified according to Heath and Packer [18], using a thiobarbituric acid (TBA) reagent. Freshly harvested leaf tissue (approximately 0.5 g) was macerated with 3 mL of 5% (w/v) trichloroacetic acid (TCA) and centrifuged at 11,500 $\times$g. A 1 mL volume of the supernatant was combined with TBA reagent (0.5% 4 mL TBA; 20% TCA) and incubated for 30 min at 95 ℃ in a water bath. After cooling, the absorbance at 532 nm was read, followed by a second reading at 600 nm for nonspecific values. After subtracting the nonspecific values, the final MDA content was determined utilizing an extinction coefficient of 155 mM^− 1^ cm^− 1^ and expressed as nmol g^− 1^ FW.

### Determination of hydrogen peroxide levels

The H_2_O_2_ content was estimated by adding 3 mL of 5% TCA to freshly harvested leaf samples (approximately 0.5 g) and centrifuging at 11,500 $\times$g at 4 °C. A 1 mL sample of the supernatant was combined with potassium iodide (1 mL) and potassium phosphate buffer (1 mL) (pH 7.0) and placed in the dark at room temperature for 1 h. The absorbance at 390 nm was then read, and the H_2_O_2_ content was expressed as nmol g^− 1^ FW [19].

### Quantification of electrolyte leakage

Electrolyte leakage (EL) was quantified as described by Dionisio-Sese and Tobita [20]. A freshly harvested leaf sample (0.5 g) was cut into small pieces, transferred to a Falcon tube containing 15 mL of dH_2_O, and incubated in a 40 °C in a water bath for 60 min. After cooling the solution to room temperature, the electrical conductivity (EC_1_) was monitored using an EC meter. The Falcon tubes were then heated again at 121 °C, and a second EC reading (EC_2_) was taken after the cooling procedure. The EL was determined using the following formula:

$$Electrolyte{\text{ }}leakage{\text{ }}\% = \frac{{EC1}}{{EC2}} \times 100$$

### Estimation of leaf proline content

The protocol described by Bates et al. [21] was used for proline (Pro) quantification. A leaf sample (approximately 0.5 g) was homogenized with an ice-cooled mortar and pestle in 5 mL of 3% aqueous sulfosalicylic acid and centrifuged at 11,500 $\times$g for 15 min at 4 °C. A 1 mL sample of the supernatant was then combined with 1 mL of ninhydrin reagent (glacial acetic acid and acid ninhydrin dissolved in 6 M phosphoric acid) and heated for 1 h in a 100 °C water bath. After the solution had cooled to room temperature, the Pro was extracted from the aqueous solution by adding 4 mL of toluene. The absorbance of the toluene layer was then read at 520 nm, and the Pro content was calculated against a standard curve of known Pro concentrations.

### Measurement of ascorbate and glutathione pools

The protocol of Kampfenkel et al. [22] was used to quantify the content of the nonenzymatic antioxidants ascorbic acid (AsA) and glutathione (GSH) in freshly harvested leaf samples (0.5 g). The leaf samples were homogenized in 3 mL of 1 mM ethylenediaminetetraacetic acid (EDTA) in 5% meta-phosphoric acid and centrifuged at 11,500 $\times$g at 4 °C. The AsA-GSH pool activities were estimated by neutralizing an aliquot with 0.5 M potassium phosphate buffer (pH 7.0) containing 0.1 M dithiothreitol (DTT) and adding dH_2_O to determine the total AsA and reduced AsA contents. The neutralized aliquot was mixed with 100 mM potassium phosphate buffer (pH 6.5) and 0.5 units of ascorbate oxidase (AO), and the absorbance was read at 265 nm in a spectrophotometer. The amounts of total AsA and reduced AsA were determined by comparison to a standard curve of known AsA concentrations, and dehydroascorbate (DHA) amounts were quantified by subtracting the amounts of reduced AsA from the total AsA [23].

Another aliquot of 10 µM supernatant was then neutralized with 0.5 M potassium phosphate buffer (pH 7.0), oxidized with 5,5-dithio-bis (2-nitrobenzoic acid) (DTNB), and then reduced with nicotinamide adenine dinucleotide phosphate (NADPH) in the presence of glutathione reductase (GR). The GSH levels were determined by reading the absorbance at 412 nm. The oxidized glutathione (GSSG) levels were determined by neutralizing the extract with 2-vinylpyridiene in potassium phosphate buffer. The contents of GSG and GSSG were estimated using a standard curve containing 12, 16, 20, and 24 µg mL^− 1^ GSH in 5% metaphosphoric acid, and then subtracting the values of GSSG from the total GSH to yield the GSH content [24].

### Assays of antioxidant enzyme activities

A 0.5 g sample of fresh leaf tissue was harvested, homogenized in extraction buffer (1 mL) containing 50 mM potassium phosphate buffer (pH 7.0), 100 mM KCl, 5 mM β-mercaptoethanol, 1 mM L-ascorbic acid, and 10% (w/v) glycerol in an ice-cooled mortar and pestle, and then centrifuged at 11,500 $\times$g for 15 min at 4 °C.

The protein content was determined using the Bradford method [25] by combining 5 µL of supernatant with 5 mL of Bradford reagent and measuring the absorbance at 595 nm, followed by comparison to a standard curve constructed containing known concentrations of bovine serum albumin. The supernatant was used as the protein extract for all enzyme assays and was maintained at 4 °C. The ascorbate peroxidase (APX; EC, 1.11.1.11) activity was measured using the protocol of Nakano and Asada [26] by adding protein extract to a reaction mixture containing 15 mM potassium phosphate buffer (pH 7.0), 0.1 mM H_2_O_2_, 0.5 mM L-ascorbic acid, and 0.1 mM EDTA. The absorbance was then read at 290 nm, and an extinction coefficient (2.8 mM^− 1^ cm^− 1^) was used to estimate the APX enzyme activity.

The method of Hossain et al. [27] was used to determine the activity of monodehydroascorbate reductase (MDHAR, EC: 1.6.5.4). An aliquot of protein extract was added to the reaction mixture containing 50 mM Tris-HCl buffer (pH 7.5), 1 unit of AO, 0.2 mM NADPH, and 2.5 mM L-ascorbic acid. The absorbance was read at 340 nm, and the MDHAR activity was estimated using an extinction coefficient of 6.2 mM^− 1^ cm^− 1^.

The method of Nakano and Asada [26] was followed to determine dehydroascorbate reductase (DHAR, EC:1.8.5.1) activity by adding protein extract to a reaction mixture containing EDTA, potassium phosphate buffer (pH 7.0), DHA, and GSH. The absorbance was read at 265 nm, and the activity of the DHAR was determined using an extinction coefficient of 14 mM^–1^ cm^–1^.

The activity of glutathione reductase (GR, EC:1.6.4.2) was estimated by adding protein extract to a reaction mixture containing 0.1 M potassium phosphate buffer (pH 7.8), 1 mM oxidized GSSG, 0.2 mM NADPH, and 1 mM EDTA. The absorbance was read at 340 nm, and the activity was determined using an extinction coefficient of 6.2 mM^− 1^ cm^− 1^ [24].

Catalase (CAT, EC: 1.11.1.6) activity was assessed by adding protein extract to a reaction mixture containing 50 mM potassium phosphate buffer (pH 7.0) and 15 mM H_2_O_2_ and determining the decrease in absorbance at 240 nm, utilizing an extinction coefficient of 39.4 M^− 1^ cm^− 1^ [24].

Glutathione peroxide (GPX, EC:1.11.1.9) activity was determined using the protocol of Nahar et al. [28] by adding 10 µL of protein extract to a reaction mixture containing 1 mM EDTA, 1 mM sodium azide, 0.12 mM NADPH, 2 mM GSH, 1 unit of GR, potassium phosphate buffer (pH 7.0), and H_2_O_2_. The activity was determined by measuring the absorbance at 340 nm and using an extinction coefficient of 6.62 mM^− 1^ cm^− 1^.

The lipoxygenase (LOX, EC: 1.13.11.12) activity was determined using the protocol of Doderer et al. [29] by adding protein extract to a reaction mixture containing sodium phosphate buffer (pH 6.5) containing Tween-20 and linolenic acid substrate. The activity was determined by reading the absorbance at 234 nm and using an extinction coefficient of 25 mM^− 1^ cm^− 1^.

The activity of glutathione-*S*-transferase (GST, EC: 2.5.1.18) was determined using the protocol of Hasanuzzaman et al. [24] by adding protein extract to a reaction mixture containing 0.25 M potassium phosphate buffer (pH 6.5), 1.5 mM GSH, and 1 mM 1-chloro-2,4-dinitrobenzene. The activity was determined by measuring the absorbance at 340 nm and using an extinction coefficient of 9.6 mM^− 1^ cm^− 1^.

The protocol of El-Shabrawi et al. [30] was used to determine the activity of superoxide dismutase (SOD, EC:1.15.1.1) by adding 5 µL of protein extract to a reaction mixture containing 50 mM potassium phosphate buffer (pH 7.0), 2.24 mM nitro blue tetrazolium chloride, 2.36 mM xanthine, and xanthine oxidase. The activity was determined by measuring the absorbance at 560 nm.

The activity of peroxidase (POD, EC: 1.11.1.7) was estimated using the protocol of Hemeda et al. [31] by adding protein extract to a reaction mixture containing 1.5 mM guaiacol as the organic substrate, 0.5 M potassium phosphate buffer (pH 7.0), and 30 mM H_2_O_2_. The activity was determined by measuring the absorbance at 470 nm with a spectrophotometer and utilizing an extinction coefficient of 26.6 mM^− 1^ cm^− 1^.

### Estimation of methylglyoxal content and glyoxalase enzyme activities

A 250 mg leaf sample was extracted using 5% perchloric acid and centrifuged at 11,500 ×g for 12 min at 4 °C [32]. Activated charcoal was added, the sample was centrifuged again, and the supernatant was neutralized with saturated sodium carbonate solution. The neutralized sample was mixed with sodium phosphate buffer (pH 7.0) containing 0.5 M *N*-acetyl-L-cysteine and reacted for 15 min. The absorbance was measured at 288 nm the methylglyoxal (MG) content was determined by comparison to a standard curve of known MG concentrations. The activity of glyoxalase I (Gly I, EC: 4.4.1.5) was assayed following the protocol of Hasanuzzaman et al. [24] by adding the protein extract to a reaction mixture containing 100 mM potassium phosphate buffer (pH 7.0), 16 mM magnesium sulfate, 35 mM MG, and 100 mM GSH. The enzyme activity was determined by measuring the absorbance at 240 nm and using an extinction coefficient of 3.37 mM^− 1^ cm^− 1^. Similarly, glyoxalase II (Gly II, EC: 3.1.2.6) activity was assayed by adding the protein extract to a reaction mixture containing 100 mM Tris-HCl buffer (pH 7.2), 0.2 mM DTNB, and 1 mM *S*-D-lactoylglutathione. The activity was determined by measuring the absorbance at 412 nm and using an extinction coefficient of 13.6 mM^− 1^ cm^− 1^.

### Statistical analysis

CoStat v.6.400 (2008) software was used for the analysis of variance (ANOVA) of the data obtained from the measured parameters. The mean separation was then compared using Tukey’s honest significant difference (HSD) test at the 5% level of significance.

## Results

### Foliar Se(np) and Se application improved wheat growth under drought

**Fig. 1:**
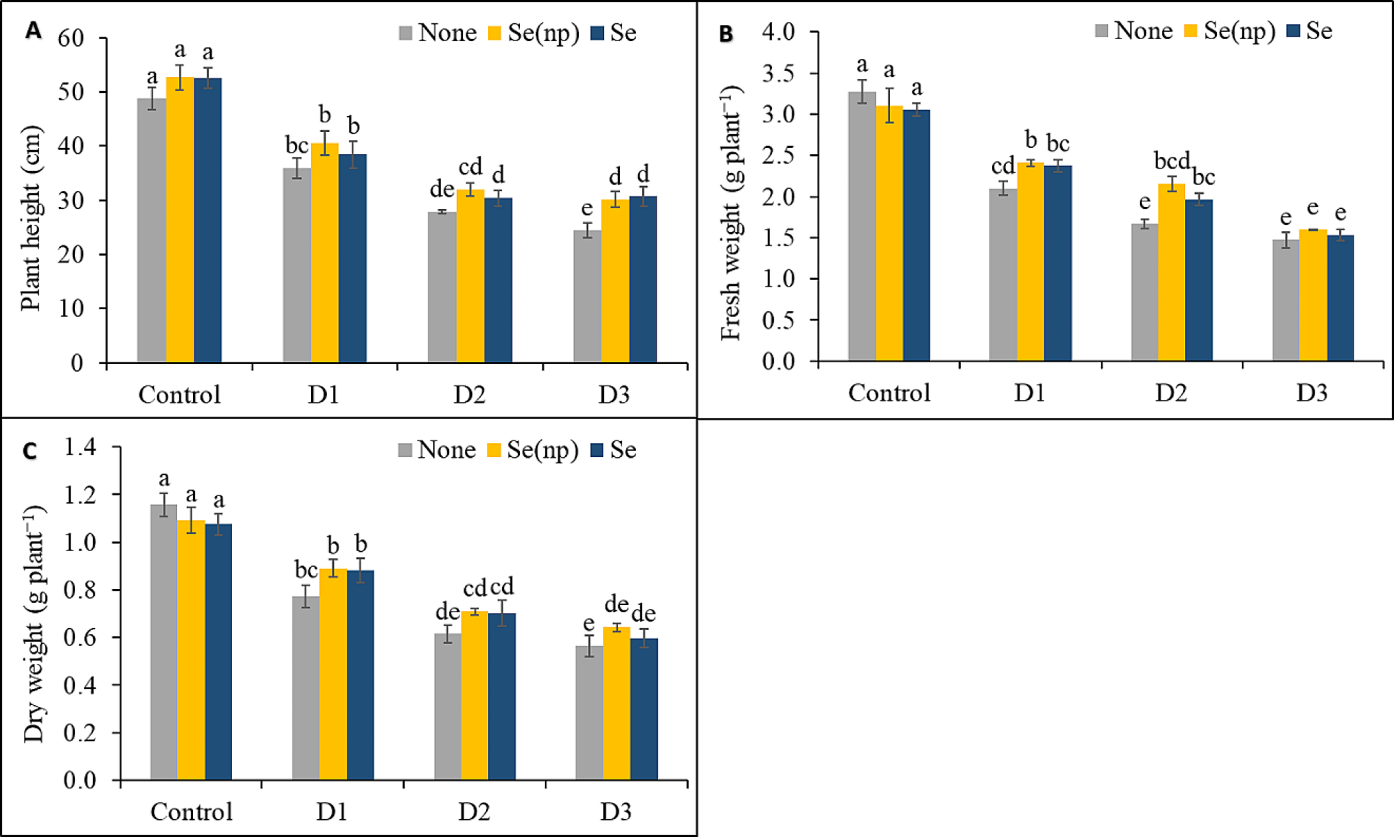
Wheat plant height **(A)**, fresh weight **(B)** and dry weight **(C)** as influenced by foliar spraying of Se(np) and Se under different water regimes [Control = well-watered; D_1_ = 50% field capacity (mild drought); D_2_ = 25% field capacity (moderate drought); D_3_ = 12.5% field capacity (severe drought)]. The values are the means ± SEMs. The means were compared by Tukey’s honestly significant difference test at *p* = 0.05. Bars with the same letter do not differ significantly from each other

Drought significantly impaired the growth of wheat, as indicated by decreases of 50%, 43%, and 26% in plant height in the D_3_, D_2_, and D_1_ drought treatments, respectively, compared to those in the unsprayed, unstressed (henceforth untreated) controls (Fig. 1A). However, the Se(np) and Se treatments negated these drought effects, and plant height increased by 23% in response to the Se(np) treatment and by 26% in response to the Se treatment compared to the unsprayed, severe (D_3_) drought-stressed controls. Similarly, compared to the untreated controls (Fig. 1B), plants subjected to the D_3,_ D_2_, and D_1_ drought regimens also showed reduced fresh weight, with reductions of 55%, 49%, and 36%, respectively. Foliar spraying with Se(np) and Se significantly improved tissue water retention, and the fresh weights of plants in the D_1_ and D_2_ drought conditions were 15% and 29% greater after Se(np) treatment and 13% and 18% greater after Se treatment, respectively, compared to the respective untreated controls.

Drought stress also significantly decreased wheat dry weight, with reductions of 33%, 47%, and 51% in the D_1_, D_2_, and D_3_ drought regimes, respectively (Fig. 1C). Dry weights were statistically similar to those of the unsprayed drought-stressed controls after foliar spraying of Se(np) and Se under all three drought conditions, although spraying tended to reduce the negative effects of drought. Under moderate drought conditions, the sprayed wheat plants attained a dry weight similar to that of the unsprayed controls under mild drought conditions. Similarly, under severe drought conditions, the dry weight of sprayed wheat plants was nearly equal to that of unsprayed plants under moderate drought conditions.

### Foliar Se(np) and Se application reduced drought-induced chlorophyll degradation

**Fig. 2:**
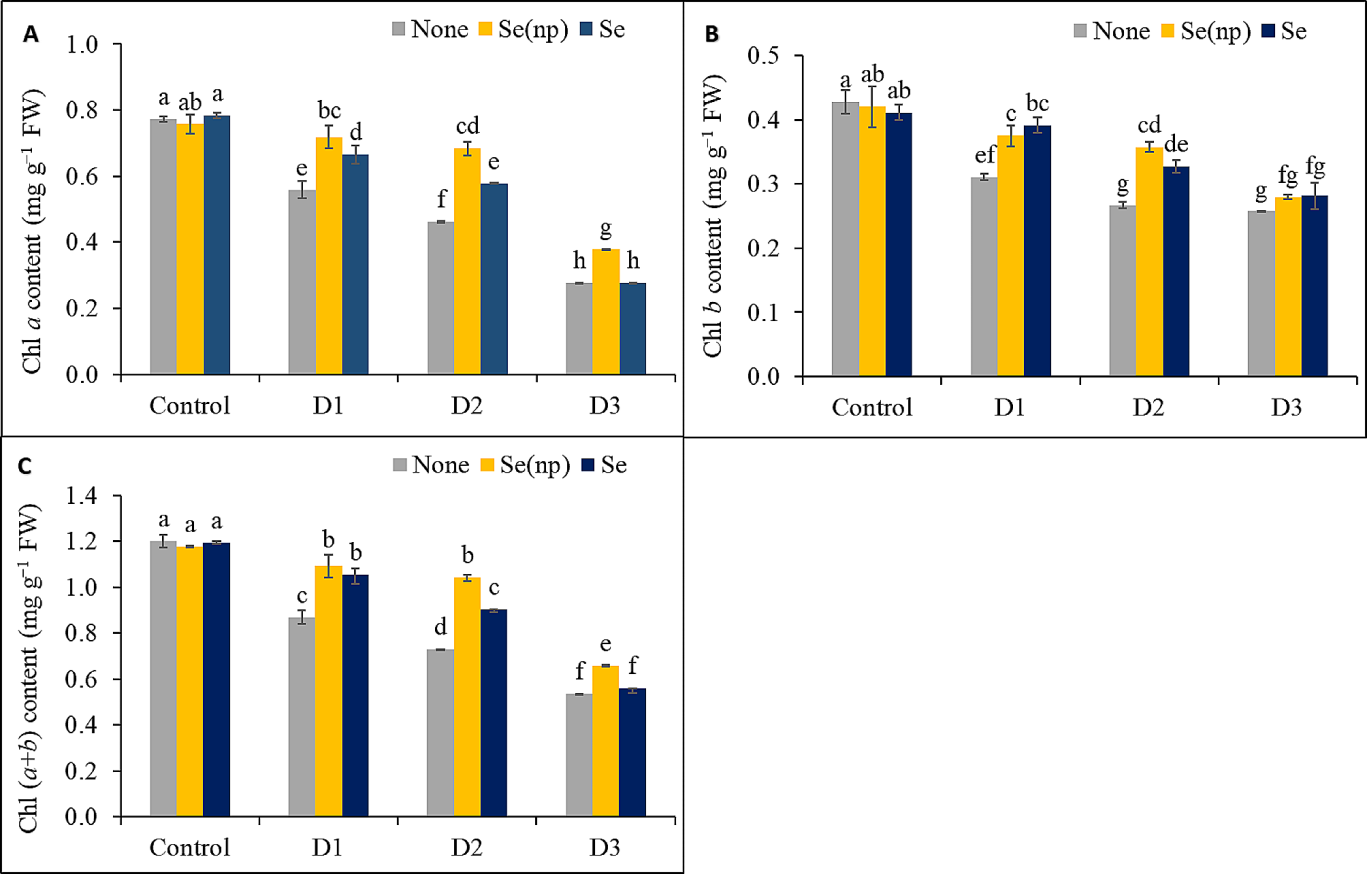
The chlorophyll *a* content **(A)**, chlorophyll *b* content **(B)** and total chlorophyll content **(C)** of wheat influenced by foliar spraying of Se(np) and Se under different water regimes [Control = well-watered; D_1_ = 50% field capacity (mild drought); D_2_ = 25% field capacity (moderate drought); D_3_ = 12.5% field capacity (severe drought)]. The values are the means ± SEMs. The means were compared by Tukey’s honestly significant difference test at *p* = 0.05. Bars with the same letter do not differ significantly from each other

The Chl *a*, Chl *b*, and total Chl contents of the wheat plants decreased significantly in response to drought. The Chl *a* content was reduced by 1.38-fold, 1.67-fold, and 2.79-fold under the D_1_, D_2_, and D_3_ drought regimes, respectively, compared to the untreated controls (Fig. 2A). However, the plants subjected to foliar spraying of Se(np) and Se application showed greater Chl *a* content under drought conditions, with Se(np)-sprayed plants showing up to 1.28-fold greater Chl levels in D_1_, 1.48-fold greater Chl levels in D_2_, and 1.36-fold greater Chl levels in D_3_ compared to the respective unsprayed drought-stressed control plants. Similarly, plants sprayed with Se had 1.19-fold and 1.25-fold greater Chl contents under D_1_ and D_2_, respectively, compared to the respective unsprayed drought-stressed controls.

The drought-induced reductions in Chl *b* were 1.38-fold, 1.61-fold, and 1.67-fold greater under D_1_, D_2_, and D_3_, respectively, compared to the untreated controls (Fig. 2B). Plants sprayed with Se(np) showed 1.21-fold and 1.34-fold higher Chl *b* levels under the D_1_ and D_2_ conditions, respectively, compared to the unsprayed drought-treated controls. Plants treated with Se also showed Chl *b* levels that were 1.26-fold and 1.22-fold higher in the D_1_ and D_2_ treatment groups, respectively, than in the respective unsprayed drought-stressed plants.

The decreases in Chl *a* and Chl *b* were also reflected in the total Chl content, which declined by 1.38-fold, 1.69-fold, and 2.25-fold, respectively, in unsprayed plants grown under the D_1_, D_2_, and D_3_ conditions (Fig. 2C). Plants sprayed with Se(np) showed 1.26-fold, 1.43-fold, and 1.23-fold higher total Chl levels in the D_1_, D_2_, and D_3_ treatment groups than in the unsprayed drought-stressed control groups, respectively. Plants sprayed with Se showed 1.21- and 1.24-times higher total Chl levels under D_1_ and D_2_, respectively, compared to the respective untreated controls.

### Foliar Se(np) and Se application reduced oxidative stress and protected membrane integrity

**Fig. 3:**
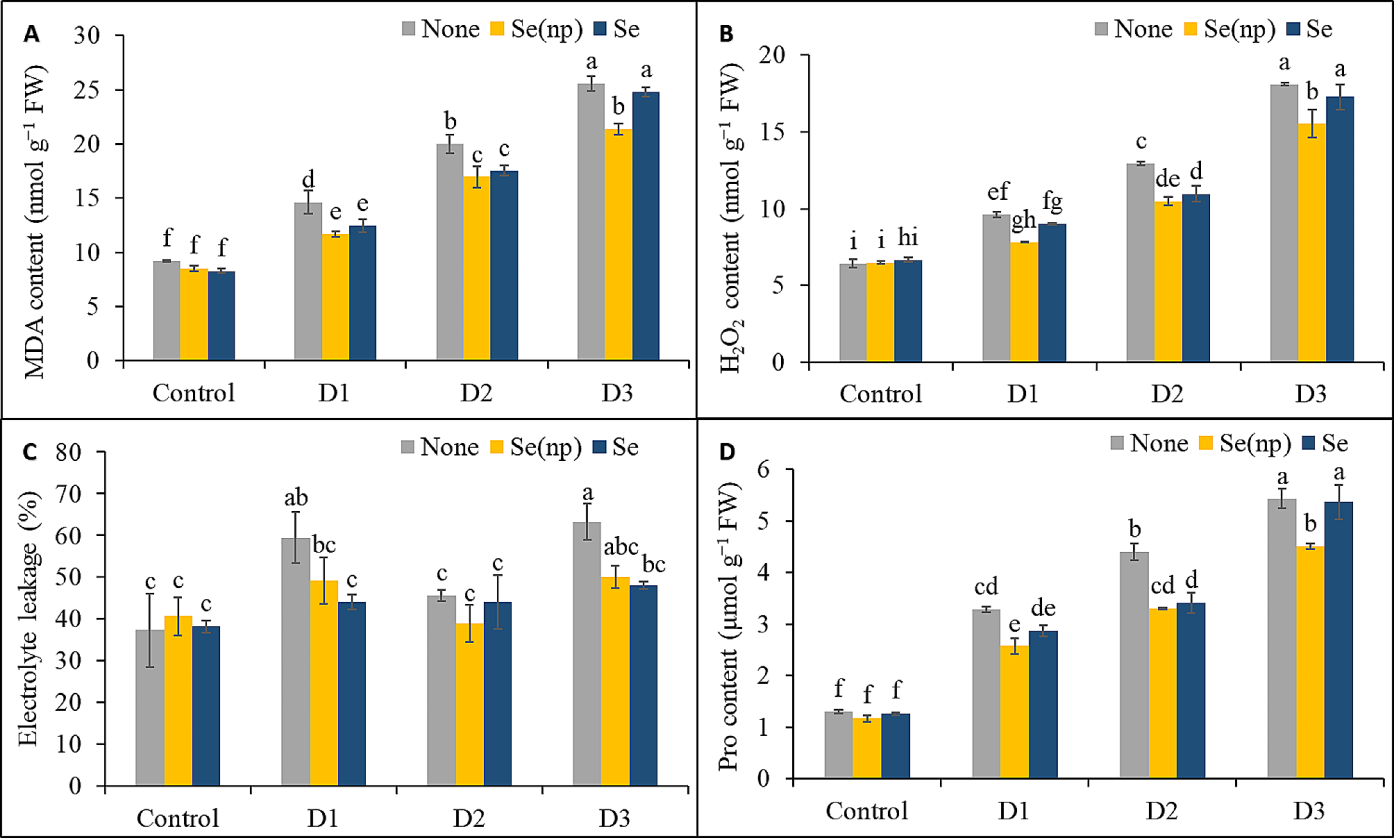
The MDA content **(A)**, H_2_O_2_ content **(B)**, electrolyte leakage **(C)** and proline content **(D)** of wheat influenced by foliar spraying of Se(np) and Se under different water regimes [Control = well-watered; D_1_ = 50% field capacity (mild drought); D_2_ = 25% field capacity (moderate drought); D_3_ = 12.5% field capacity (severe drought)]. The values are the means ± SEMs. The means were compared by Tukey’s honestly significant difference test at *p* = 0.05. Bars with the same letter do not differ significantly from each other

Drought stress significantly increased oxidative stress in wheat leaves, as indicated by increases in the MDA and H_2_O_2_ contents. Compared with the untreated controls, the plants in the D_1_, D_2_, and D_3_ treatment groups showed increases in MDA levels of 1.59-fold, 2.17-fold, and 2.77-fold, respectively (Fig. 3A). Similarly, the H_2_O_2_ content was 1.5-fold, 2.02-fold, and 2.81-fold greater in the D_1_, D_2_, and D_3_ treatment groups, respectively than in the untreated control groups (Fig. 3B). Plants sprayed with Se(np) showed reduced oxidative damage during drought stress, as indicated by lower MDA and H_2_O_2_ contents under the D_1_, D_2_, and D_3_ water regimes compared to the untreated control plants. By contrast, plants sprayed with Se showed reduced MDA levels under the D_1_ and D_2_ conditions and lower H_2_O_2_ contents under D_2_ conditions when compared with the respective unsprayed drought-stressed plants.

Drought stress also impaired plant membrane stability, as indicated by 22% and 26% increases in EL under the D_1_, and D_3_ water regimes, respectively, compared to the untreated controls (Fig. 3C). However, plants treated with Se under drought conditions exhibited greater membrane stability, with lower electrolyte leakage in the D_1_ (15%) and D_3_ (15%) treatment groups only, respectively, than in the respective unsprayed drought-stressed control plants. On the contrary, the plants sprayed with Se(np) did not show significant changes in EL% when subjected to drought stress.

### Foliar Se(np) and Se application altered the leaf proline content

The proline levels showed a notable increase under drought conditions, which was increased 2.52-fold, 3.39-fold, and 4.18-fold in the plants treated with D_1_, D_2_, and D_3_ drought stress, respectively, compared to those in the untreated controls (Fig. 3D). Plants treated with Se(np) showed lower accumulation of proline under the D_1_ (22%), D_2_ (25%), and D_3_ (17%) water regimes, respectively, than did their respective unsprayed drought-stressed controls. Plants treated with Se showed a 22% lower proline content in the D_2_ treatment group than in the respective unsprayed drought-stressed control group.

### Foliar Se(np) and Se application enhanced ascorbate–glutathione cycle activity

**Fig. 4:**
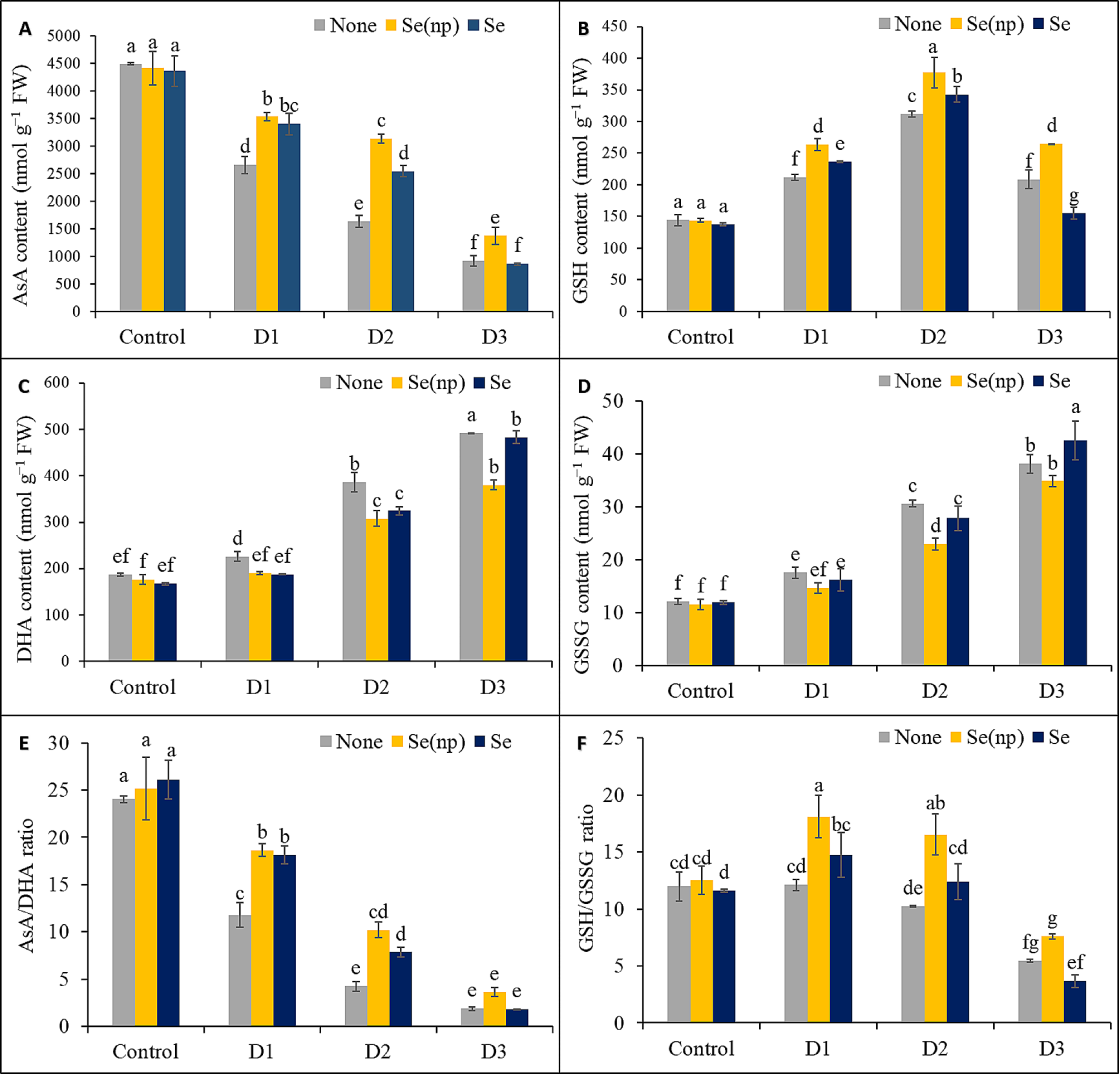
The AsA content **(A)**, GSH content **(B)**, DHA content **(C)**, GSSG content **(D)**, AsA/DHA ratio **(E)** and GSH/GSSG ratio **(F)** of wheat as influenced by foliar spraying of Se(np) and Se under different water regimes [Control = well-watered; D_1_ = 50% field capacity (mild drought); D_2_ = 25% field capacity (moderate drought); D_3_ = 12.5% field capacity (severe drought)]. The values are the means ± SEMs. The means were compared by Tukey’s honestly significant difference test at *p* = 0.05. Bars with the same letter do not differ significantly from each other

Drought stress enhanced the conversion of AsA to DHA, as plants showed 51%, 82%, and 92% lower AsA/DHA ratios under the D_1_, D_2_, and D_3_ conditions, respectively, than did the untreated controls (Fig. 4E), whereas the DHA contents were increased by 21%, 106%, and 163%, and the AsA contents were decreased by 41%, 64%, and 80%, respectively, under the D_1_, D_2_, and D_3_ water regimes, compared to the untreated controls (Fig. 4A). Plants sprayed with Se(np) showed 33%, 92%, and 49% higher AsA levels and 16%, 20%, and 23% lower DHA levels under D_1_, D_2_, and D_3_, respectively (Fig. 4A, C), as well as 58% and 140% higher AsA/DHA ratios under D_1_ and D_2_, respectively, compared to their respective unsprayed drought-stressed controls (Fig. 4E). Plants sprayed with Se had 28% and 56% greater AsA contents, 17% and 16% lower DHA contents, and 54% and 85% greater AsA/DHA ratios under D_1_ and D_2_ conditions, respectively, than the respective unsprayed drought-stressed controls. Spraying of Se(np) or Se under the D_3_ condition did not result in noticeable changes in the AsA/DHA ratio.

Plants exposed to the D_1_, D_2_, and D_3_ water regimes showed 47%, 116%, and 45% higher levels of GSH and 45%, 153%, and 215% higher levels of GSSG, respectively, compared to the untreated controls (Fig. 4B, D) The ratio of GSH/GSSG was notably reduced by 54% under the D_3_ water regime compared to the untreated controls (Fig. 4F). Plants treated with Se(np) showed 25%, 21%, and 27% greater GSH contents under D_1_, D_2_, and D_3_, respectively, as well as a 25% lower GSSG content under D_2_ compared to the respective unsprayed drought-stressed controls. The plants sprayed with Se(np) also had 50% and 62% greater GSH/GSSG ratios under D_1_ and D_2_, respectively, than the unsprayed drought-stressed controls. Moreover, plants sprayed with Se showed 26% lower GSH levels under D_3_ conditions but 12% and 10% greater GSH levels under D_1_ and D_2_ conditions, respectively, than the drought-stressed controls. Plants sprayed with Se also had 12% greater GSSG content in the D_3_ treatment group than in the respective unsprayed drought-stressed control group. However, the ratio of GSH/GSSG did not change notably in the Se-sprayed plants in the D_1_, D_2_, and D_3_ drought-stressed groups.

### Foliar Se(np) and Se applications upregulate antioxidant enzymes

**Fig. 5:**
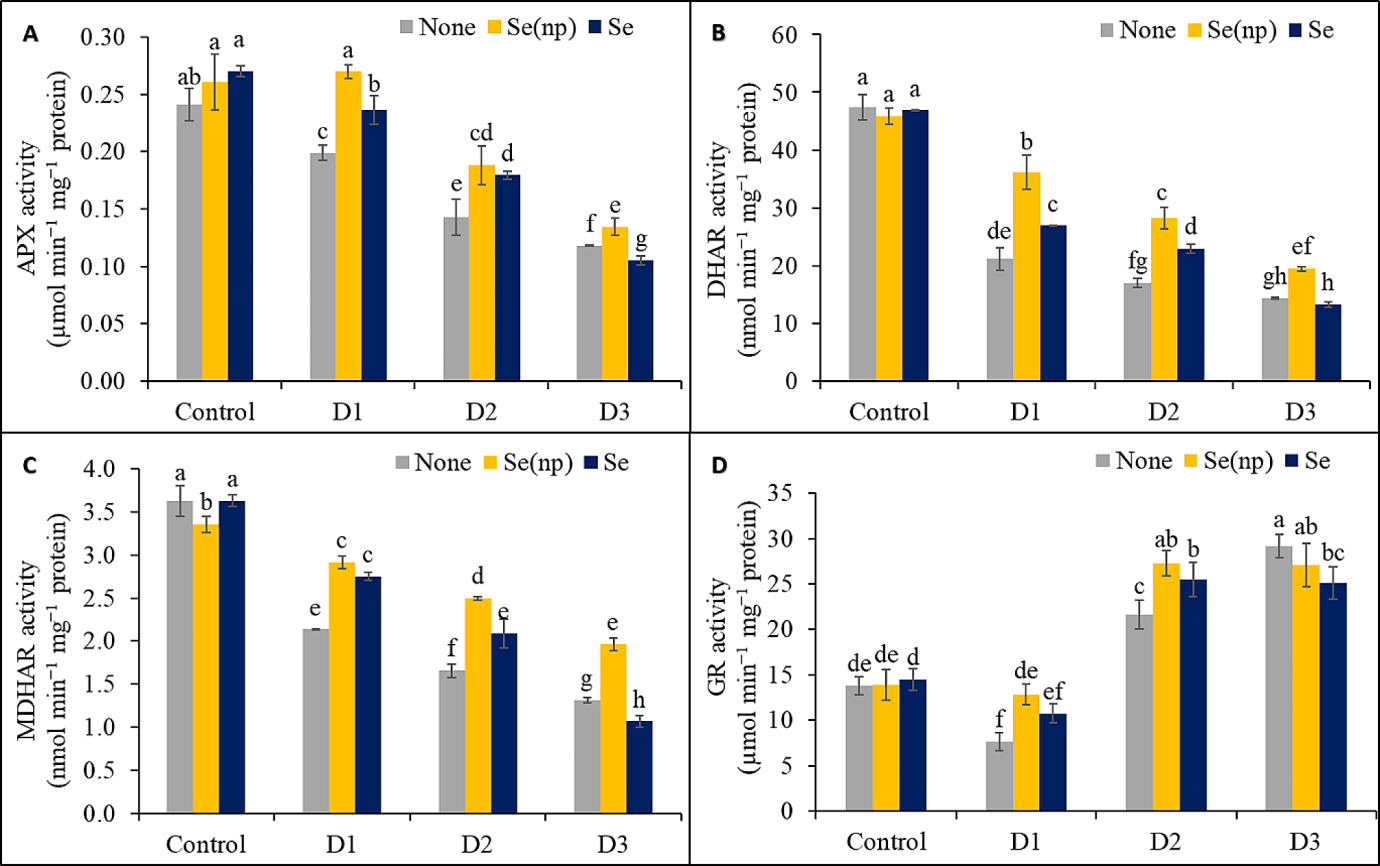
The APX activity **(A)**, DHAR activity **(B)**, MDHAR activity **(C)** and GR activity **(D)** of wheat as influenced by foliar spraying of Se(np) and Se under different water regimes [Control = well-watered; D_1_ = 50% field capacity (mild drought); D_2_ = 25% field capacity (moderate drought); D_3_ = 12.5% field capacity (severe drought)]. The values are the means ± SEMs. The means were compared by Tukey’s honestly significant difference test at *p* = 0.05. Bars with the same letter do not differ significantly from each other

Unsprayed plants showed 17%, 41%, and 51% lower APX activity (Fig. 5A), 55%, 64%, and 70% lower DHAR activity (Fig. 5B), and 41%, 54%, and 64% lower MDHAR activity (Fig. 5C) under the D_1_, D_2_, and D_3_ conditions, respectively, than their respective untreated controls. Compared to the untreated controls, the unsprayed drought-stressed plants also exhibited 45% lower GR activity under the D_1_ condition but 57% and 112% greater GR activity under the D_2_ and D_3_ conditions, respectively (Fig. 5D).

Plants sprayed with Se(np) under the D_1_, D_2_, and D_3_ water regimes showed a notable increase in APX activity (by 36%, 32%, and 14%, respectively), DHAR activity (by 71%, 66%, and 36%, respectively), and MDHAR activity (by 36%, 51%, and 50%, respectively) compared to the respective unsprayed drought-stressed controls, whereas GR activity was increased by 68% and 26% in the D_1_ and D_2_ conditions compared to the respective unsprayed drought-stressed controls. Plants sprayed with Se showed 19% and 26% greater APX activity, 27% and 34% greater DHAR activity, 29% and 26% greater MDHAR activity, and 41% and 18% greater GR activity under D_1_ and D_2_, respectively, compared to the respective unsprayed drought-stressed controls. Notably, under D_3_ conditions, plants sprayed with Se had 11% lower APX activity, 19% lower MDHAR activity, and 14% lower GR activity than the respective unsprayed drought-stressed controls.

**Fig. 6:**
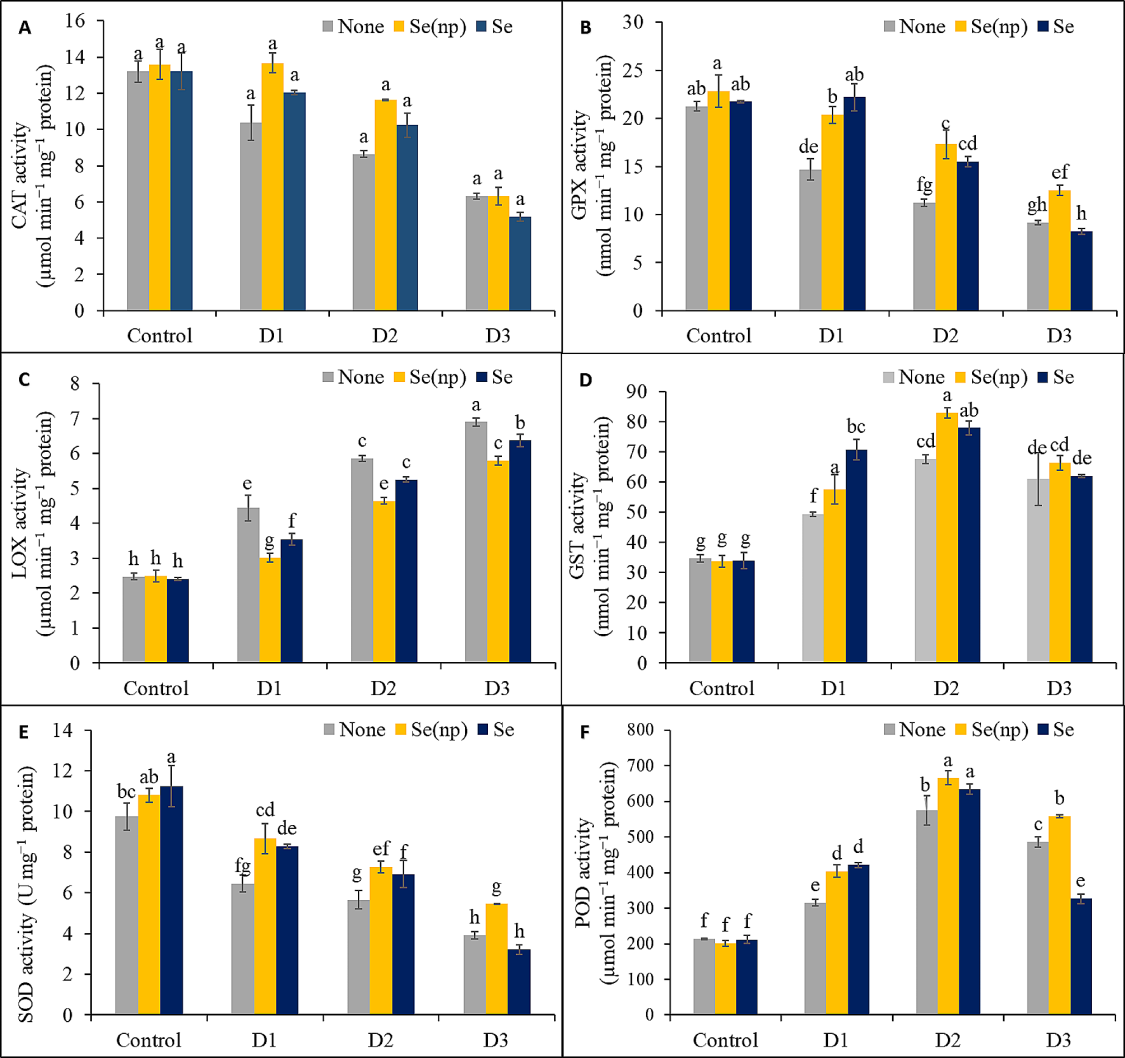
The CAT activity **(A)**, GPX activity **(B)**, LOX activity **(C)**, GST activity **(D)**, SOD activity **(E)** and POD activity **(F)** of wheat as influenced by foliar spraying of Se(np) and Se under different water regimes [Control = well-watered; D_1_ = 50% field capacity (mild drought); D_2_ = 25% field capacity (moderate drought); D_3_ = 12.5% field capacity (severe drought)]. The values are the means ± SEMs. The means were compared by Tukey’s honestly significant difference test at *p* = 0.05. Bars with the same letter do not differ significantly from each other

The activity of CAT was not significantly influenced by the different drought regimes (Fig. 6A). Drought decreased GPX activity by 31%, 47%, and 57% (Fig. 6B), and SOD activity by 34%, 42%, and 60% (Fig. 6E), respectively, in the D_1_, D_2_, and D_3_ water regimes compared to the unsprayed drought-stressed controls. On the contrary, the LOX activity was increased by 1.79-fold, 2.37-fold, and 2.78-fold, the GST activity by 1.42-fold, 1.95-fold, and 1.75-fold, and POD activity by 1.48-fold, 1.7-fold, and 2.28-fold, respectively, in the D_1_, D_2_, and D_3_ conditions than in the untreated controls (Fig. 6C and D, and 6F). Spraying of Se(np) increased the activities of GPX, SOD, and POD in the D_1_, D_2_, and D_3_ treatment groups and the GST activity in the D_1_ and D_2_ conditions, whereas the activity of LOX was decreased in the D_1_, D_2_, and D_3_ treatment groups compared to the drought stress alone. Moreover, Se treatment increased the GPX, GST, SOD, and POD activities under mild (D_1_) and moderate (D_2_) drought stress, and reduced the LOX activity in the D_1_, D_2_, and D_3_ conditions compared to the unsprayed drought-stressed plants.

### Foliar Se(np) and Se application altered glyoxalase system activity

**Fig. 7:**
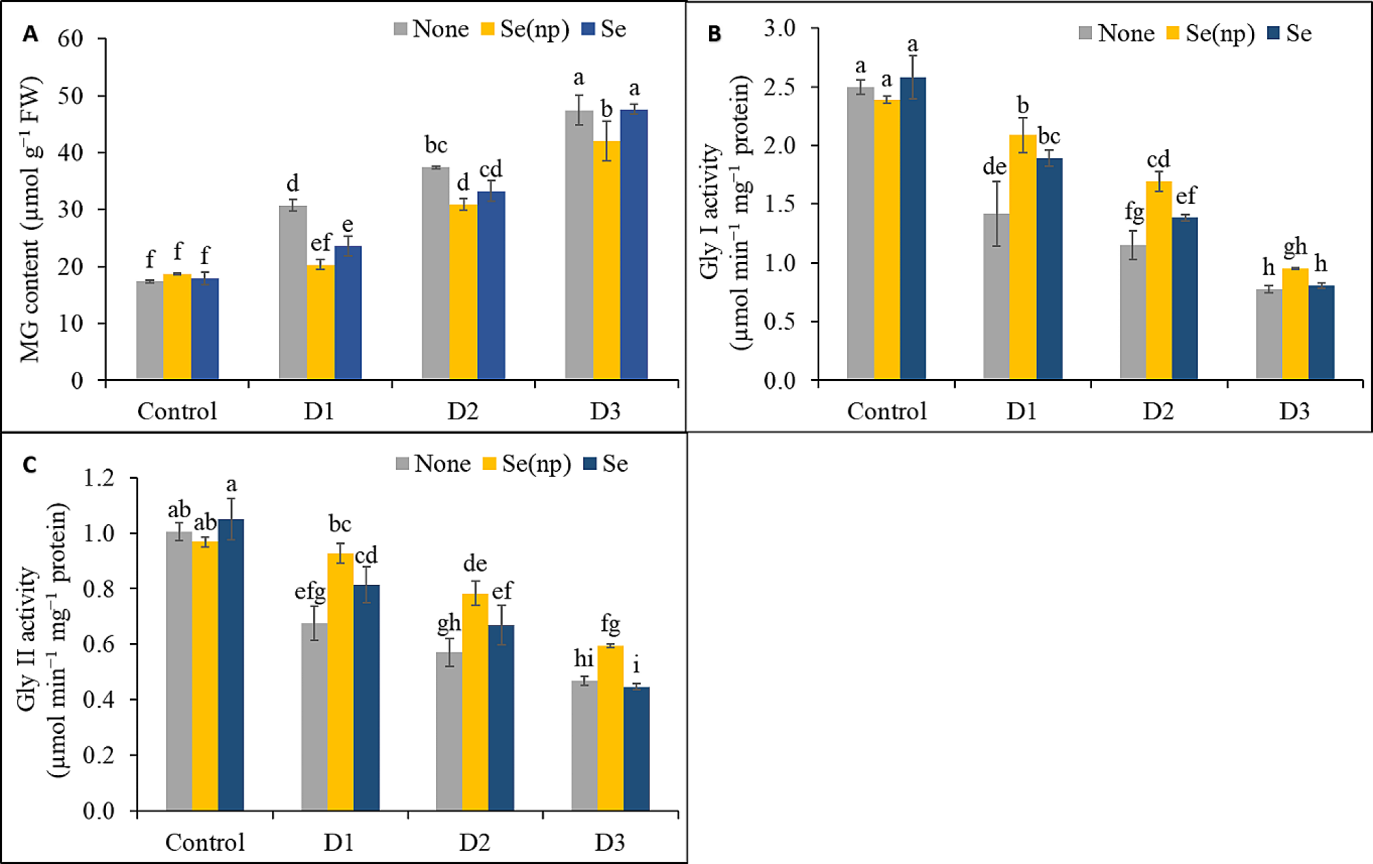
The MG content **(A)**, Gly I activity **(B)** and Gly II activity **(C)** of wheat as influenced by foliar spraying of Se(np) and Se under different water regimes [Control = well-watered; D_1_ = 50% field capacity (mild drought); D_2_ = 25% field capacity (moderate drought); D_3_ = 12.5% field capacity (severe drought)]. The values are the means ± SEMs. The means were compared by Tukey’s honestly significant difference test at *p* = 0.05. Bars with the same letter do not differ significantly from each other

Plants exposed to drought showed 1.77-fold, 2.16-fold, and 2.73-fold higher MG contents (Fig. 7A), but 1.76-fold, 2.17-fold, and 3.23-fold lower activities of Gly I (Fig. 7B) and 1.49-fold, 1.76-fold, and 2.14-fold lower activities of Gly II (Fig. 7C) in the D_1_, D_2_, and D_3_ conditions, respectively, compared to the untreated controls. However, spraying with Se(np) under the D_1_ and D_2_ conditions increased the Gly I and Gly II activities, but reduced the MG levels in the D_1_, D_2_, and D_3_ conditions compared to the unsprayed drought-stressed plants. Plants sprayed with Se also showed a similar trend of reduction of MG levels under the D_1_ and D_2_ treatment, increased Gly I activity in the D_1_ condition, and increased Gly II activity in the D_1_ and D_2_ conditions compared to the respective unsprayed drought-stressed controls.

## Discussion

Plant growth processes are negatively impacted by water deficit conditions or drought stress due to the resulting disruptions in cytosolic metabolism, reductions in assimilate production, suppression of vegetative growth and development, and decreases in productivity. Drought stress reduces the photosynthesis rate in several ways—by altering the levels of photosynthetic pigments in leaves, decreasing leaf area, reducing stomatal conductivity, enhancing lipid peroxidation in cell membranes, and suppressing protein and Chl synthesis—with deleterious effects on plant growth [33]. In the present study, the growth of wheat plants was clearly decreased under drought conditions. Similar decreases in plant biomass (dry weight and fresh weight) have been previously reported in many plants, including *Salvia officinalis* L. by Ostadi et al. [34] and *Lallemantia iberica* by Javanmard et al. [35], when grown under mild and moderate drought stress. These declines in plant growth attributes have increased interest in the use of chemical elicitors and nanotechnology to improve plant growth and development under abiotic stresses, such as water stress.

In the present study, foliar applications of Se and Se(np) clearly mitigated the negative effects of drought stress on wheat growth and development. Several studies have demonstrated positive effects of Se on growth parameters and the enhancement of dry matter accumulation, plant height, and leaf area in drought-affected plants. A previous study also revealed improved plant growth under drought in wheat seedlings of two different genotypes after treatment with a foliar Se spray [36]. Overall, the use of Se and Se(np) appears to accelerate plant root growth, thereby enhancing nutrient absorption from the soil and improving the rates of photosynthesis, with a consequent suppression of the adverse effects of stress on plant growth characteristics [37].

In the present study, different levels of drought stress significantly reduced the Chl pigment content of wheat leaves. This reduction enhanced ROS generation and subsequent membrane damage, eventually leading to the further destruction of leaf Chl pigments [38]. A reduction in photoassimilate production would also limit the energy needed for the absorption of nutrients (e.g., Mg) from the soil, which would also affect Chl biosynthesis [39]. However, foliar sprays of Se and Se(np) restored Chl pigment contents in drought-stressed wheat plants in this study. This effect might be a result of the Se-induced suppression of membrane lipid peroxidation in plants. Seliem et al. [40] reported a similar increase in leaf Chl *a* and Chl *b* contents in *Chrysanthemum morifolium* in response to Se(np) application. Other studies using Se(np), including experiments on *Solanum lycopersicum* by Haghighi et al. [41] and *Vigna unguiculata* L. by El Lateef Gharib et al. [15], have reported that Se(np) are more effective than Se alone in improving Chl pigment levels, in agreement with the findings of the present study. Higher rates of CO_2_ assimilation, enhanced rates of photosynthesis, and improved soil nutrient uptake are the supposed causes of these Se(np) effects [15].

Another possible mechanism that could explain Se(np) effects may involve H_2_O_2_ production. Although H_2_O_2_ is an important signaling molecule, its levels become elevated during oxidative stress to defend against environmental stresses. The generation of ROS also triggers lipid peroxidation in the plasma membrane, with negative effects on cellular functioning that further increase oxidative stress. The excessive production of H_2_O_2_ and MDA can therefore serve as indicators of oxidative stress generated by various abiotic stresses in plants. In the present study, the H_2_O_2_ and MDA levels were notably elevated in response to all three levels of drought stress, confirming that plants need external elicitors to activate the antioxidant defense systems needed to supplement their innate antioxidant enzymes in times of stress. Possibly, the Se and Se(np) foliar sprays decreased the generation of H_2_O_2_ and MDA to levels below those observed in the unsprayed drought-stressed plants through the activation of antioxidant enzymes, in agreement with numerous previous results [10, 42–44]. Se itself cannot scavenge H_2_O_2_; instead, it facilitates the reduction of the cellular H_2_O_2_ content by other H_2_O_2_ quenchers [10]. Similarly, Pro [44, 45] and leaf EL% [40, 46] were reduced in plants growing in stressful environments after Se(np) and Se supplementation, in agreement with the findings of the present experiment. Foliar application of Se and Se(np) reduced wheat leaf EL% and Pro content under different levels of drought stress, indicating stress alleviation. This effect presumably arose because Se and Se(np) maintained the structural integrity of cells by enhancing ROS scavenging, ensuring proper fatty acid content, and triggering amino acid regulation of the TCA cycle to enhance lipid metabolism [46].

The deleterious effects of abiotic stresses are observed if ROS generation surpasses the capability of the antioxidant mechanisms that deal with ROS generation during normal metabolism. In this case, oxidative stress occurs in plant cells, and integrated efforts are made to activate enzymatic and nonenzymatic antioxidant activities, which are part of the antioxidant defense mechanisms required under these circumstances [47]. The APX enzyme creates MDHA as part of the nonenzymatic antioxidant pool of AsA-GSH, where AsA provides electrons. This reduction in MDHA generates DHA, which is subsequently oxidized to AsA with the help of GSH (another electron donor) under stressful conditions [48]. Through this AsA-GSH cycle, DHA and GSSG can also confer tolerance against stress in plants. In the present study, lower AsA and higher DHA levels indicated a reduction in the AsA/DHA ratio, whereas the GSH and GSSG levels showed an opposite shift, which eventually lowered their ratio. The upregulation of DHA and GSSG in wheat under different drought conditions again indicates enhanced oxidative metabolism, but the ratios are reversed by treatment with Se and Se(np) foliar sprays. Here, the activity of GR increased after Se and Se(np) foliar sprays, eventually increasing the GSH content and decreasing the GSSG content, indicating that Se and Se(np) have the potential to mitigate drought in wheat. These results corroborate similar findings from previous experiments with Se [24, 49] and Se(np) [50, 51] in different plants.

In the AsA-GSH pathway, APX reduces H_2_O_2_ to H_2_O [50]. In wheat leaves, the APX activity decreased significantly under different levels of drought stress, but the activity notably increased after treatment with Se and Se(np). This might reflect the detoxification of intracellular H_2_O_2_ because APX has a strong affinity for H_2_O_2_. Other studies have reported similar findings of APX activation following Se and Se(np) application [52–54].

The regeneration of AsA is significantly affected by MDHAR and DHAR metabolism, and both metabolites are crucial parts of plant antioxidant defense systems. Dehydroascorbate reductase increases the level of AsA under stressful conditions to facilitate the scavenging of H_2_O_2_. Moreover, NADPH is needed for the reduction of GSSH to GSH, so its levels indirectly maintain the GSH/GSSG ratio. The wheat plants in the present study displayed a notable decrease in MDHAR and DHAR activities and concomitantly greater GR activities when exposed to the three different levels of drought stress. However, the Se- and Se(np)-treated plants showed increased levels of MDHAR, DHAR, and GR activity when exposed to the same drought stresses, clearly supporting the distinctive roles of antioxidant enzymes in mitigating drought-induced oxidative damage by maintaining AsA recycling. These findings are also corroborated by previous reports [55, 56].

The wheat plants in the present study also exhibited notably increased GST, POD, and LOX activities and decreased activities of other enzymes (e.g., CAT, GPX, and SOD) under different drought stress levels. The POD enzyme converts H_2_O_2_ to H_2_O, while the SOD enzyme detoxifies O_2_^•−^ to H_2_O_2_ [57]. Catalase subsequently turns H_2_O_2_ into H_2_O and O_2_. The effects of Se and Se(np) sprays on GST, POD, and LOX activities were notable and paralleled the reductions in H_2_O_2_ levels and lipid peroxidation, indicating that Se and Se(np) were effective at mitigating drought stress in wheat plants, in agreement with Se [53, 58–60] and Se(np) [37, 56, 61] studies on other plants under different kinds of stress. A decrease in CAT activity is important for the detoxification of peroxides, whereas SOD helps detoxify O_2_^•−^ to less toxic H_2_O_2_. During water stress, the accumulation of H_2_O_2_ might inactivate CAT, an enzyme known to undergo photoinactivation [62]. POD assists in plant respiration and changes phenols into quinines, which may help with stress reduction under stressful conditions [63].

Glyoxalase enzymes work together to detoxify the harmful effects of MG that accumulates in stressful situations [64]. Therefore, the downregulated activities of Gly I and Gly II by drought stress in this study suppressed wheat growth and development, as previously reported [55, 60]. However, foliar application of Se and Se(np) enhanced the activities of Gly I and Gly II, indicating improved detoxification of MG. This was further supported by the elevated GSH levels, because GSH recycling relies on the glyoxalase system. Previous results with Se and Se(np) treatments in stressed plants also corroborate the findings of the present study [56, 60, 65, 66].

## Conclusion

This study highlights the protective roles of selenium and its nanoparticles in alleviating the adverse effects of drought stress on wheat plants, with a focus on enhancing various morphophysiological and biochemical parameters. The application of Se(np) and Se significantly enhanced the activity of several enzymes, such as the glyoxalases Gly I and Gly II, APX, MDHAR, DHAR, GR, SOD, CAT, and POD, which are key components of plant antioxidant defense systems. This enhancement facilitated rapid ROS scavenging while suppressing MG toxicity, thereby diminishing oxidative stress in wheat plants. Selenium nanoparticles and Se foliar sprays had positive effects on wheat growth and Chl synthesis, while reducing oxidative stress through the moderation of MDA and H_2_O_2_ levels and decreasing EL% by preserving membrane integrity under drought conditions. These findings suggest that Se is an effective alleviator of drought stress that acts by modifying plant ROS-scavenging and antioxidant defense mechanisms, as well as activating the glyoxalase system, thereby enhancing plant growth and development. Notably, in most aspects, Se(np) was more effective than the Se spray at mitigating drought stress. This observation indicates a need for further research to explain the molecular mechanisms and signaling pathways by which Se(np) enhances stress tolerance in plants.

### Electronic supplementary material

Below is the link to the electronic supplementary material.

Supplementary Material 1

## Data Availability

Data are provided within the manuscript.

## Abbreviations

ANOVA: Analysis of variance
APX: Ascorbate peroxidase
CAT: Catalase
Chl: Chlorophyll
DHA: Dehydroascorbate
DTNB: 5,5-dithio-bis-(2-nitrobenzoic acid)
DTT: Dithiothreitol
EC: Electrical conductivity
EDTA: Ethylenediaminetetraacetic acid
EL: Electrolyte leakage
FW: Fresh weight
Gly: Glyoxalase
GPX: Glutathione peroxide
GR: Glutathione reductase
GSSG: Oxidized glutathione
GST: Glutathione *S*-transferase
H_2_O_2_: Hydrogen peroxide
LOX: Lipoxygenase
MDHAR: Monodehydroascorbate reductase
MG: Methylglyoxal
NADPH: Nicotinamide adenine dinucleotide phosphate
NPs: Nanoparticles
POD: Peroxidase
ROS: Reactive oxygen species
Se: Selenium
SEM: Scanning electron microscopy
SLG: S-D-lactoylglutathione
SOD: Superoxide dismutase
TBA: Thiobarbituric acid
TCA: Trichloroacetic acid
TEM: Transmission electron microscopy
