# Supplementary figures and images for: Selenium and its nanoparticles modulate the metabolism of reactive oxygen species and morpho-physiology of wheat (Triticum aestivum L.) to combat oxidative stress under water deficit conditions
Source: BMC Plant Biol. 2024 Jun 19;24:578. doi: 10.1186/s12870-024-05282-3 (PMC11186265; doi:10.1186/s12870-024-05282-3)

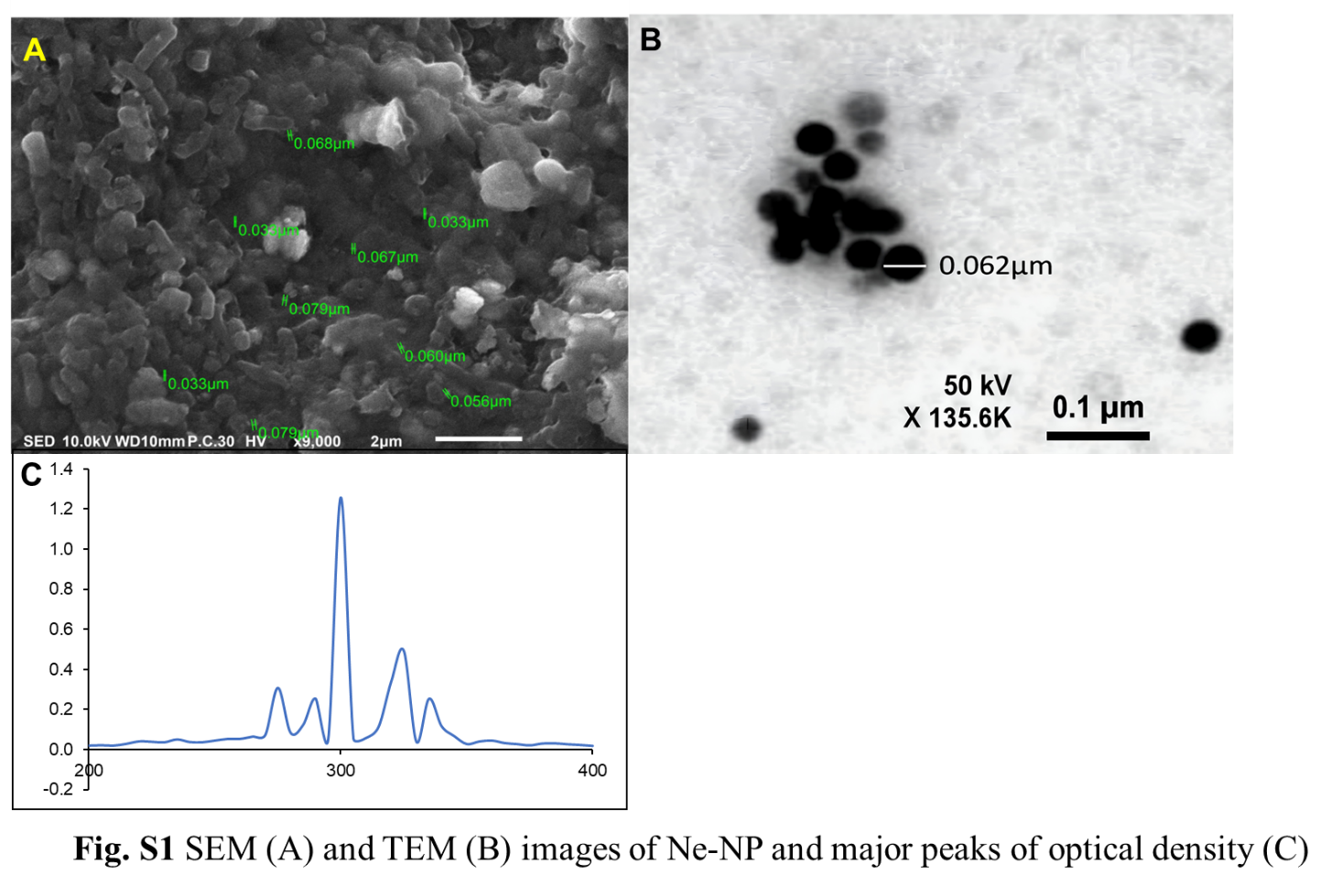


**Fig. S1** SEM (A) and TEM (B) images of Ne-NP and major peaks of optical density (C)

Supplement: Supplementary file 1 — Supplementary Material 1 [file 12870_2024_5282_MOESM1_ESM.docx]
